# Balloon occluded TACE (B-TACE) vs DEM-TACE for HCC: a single center retrospective case control study

**DOI:** 10.1186/s12876-021-01631-w

**Published:** 2021-02-03

**Authors:** Pierleone Lucatelli, Gianluca De Rubeis, Bianca Rocco, Fabrizio Basilico, Alessandro Cannavale, Aurelio Abbatecola, Pier Giorgio Nardis, Mario Corona, Stefania Brozetti, Carlo Catalano, Mario Bezzi

**Affiliations:** 1 Vascular and Interventional Radiology Unit, Department of Diagnostic Service, Sapienza University of Rome, Viale Regina Elena 324, 00161 Rome, Italy; 2 Gastroenterology Division, Department of Clinical Medicine, Sapienza University of Rome, Rome, Italy; 3 Pietro Valdoni Surgery Department, Sapienza” University of Rome, Rome, Italy

**Keywords:** Trans-catheter-arterial chemoembolization (TACE), Balloon-occluded trans-catheter arterial chemoembolization (b-TACE), Drug eluting microsphere trans arterial chemoembolization (DEM-TACE), Balloon micro-catheter, Hepatocellular-carcinoma (HCC), Safety profile, Oncological comparison

## Abstract

**Background:**

To compare oncological results and safety profile of balloon micro-catheter trans-arterial chemoembolization (b-TACE) and drug-eluting-microsphere (DEM-TACE) in patients with hepatocellular-carcinoma (HCC).

**Methods:**

This is a case–control, retrospective, single-center study. Between January-2015/March-2019, 149 patients (131 males [87.9%]) with 226 HCC were treated, 22 patients (35 HCC; 19 [86.4%] males) with b-TACE and 127 with DEM-TACE (191 HCC, 112 [88.2%] males). Embolization protocol was standardized (sequential 100 ± 25 and 200 ± 25 μm microspheres). Results were evaluated by modified-response-evaluation-criteria-in-solid-tumor [mRECIST] at 1, 3–6 and 9–12 months and time to recurrence after complete response [TTR] at 1 years. Cox’s regression weighted with tumor dimensions was performed. Adverse events (AEs) were recorded.

**Results:**

mRECIST oncological response at all time points (1, 3–6 and 9–12 months) for both treatments were similar, with the exception of Objective response rate at 9-12 months. Objective response at 1 and 3–6 months between b-TACE vs DEM-TACE [23/35 (65.7%) vs 119/191 (62.3%), 21/29 (72.4%) vs 78/136 (57.4%) (*p* > 0.05), respectively]. On the contrary, at 9–12 months, it was significantly higher in b-TACE subgroup than DEM-TACE (15/19 [78.9%] vs 48/89 [53.9%], *p* = 0.05). TTR for complete response at 1 year had a better trend for b-TACE vs DEM-TACE (278.0 days [196.0–342.0] vs 219.0 days [161.0–238.0], OR 0.68 [0.4–1.0], *p* = 0.10). The use of balloon micro-catheter reduced the relative risk of the event of recurrence by 0.63 [CI95% 0.38–1.04]; *p* = 0.07). No significant differences were found in AEs rate.

**Conclusion:**

b-TACE showed a trend of better oncological response over DEM-TACE with and longer TTR with a similar adverse events rate, in patients presenting with larger tumors.

## Background

Hepatocellular carcinoma (HCC), with definitive diagnosis (LI-RADS-5) according to Liver Imaging Reporting and Data System (LI-RADS)[1, 2] are staged according to Barcelona Clinic Liver Cancer (BCLC) staging system [3]. BCLC algorithm treatment of choice of Intermediate stage (B stage) HCC is trans-arterial chemoembolization (TACE).

Recently, the use of a balloon micro-catheter for temporary arterial occlusion has been proposed for TACE (named b-TACE procedure) [4]. The temporary arterial occlusion may enhance treatment success, due to its ability to redistribute flow towards lower resistance vascular territories (i.e. hyper-vascular HCC), thus allowing a pressure-gradient driven embolization [4] The increased accumulation of embolic particles within the tumor may lead to increased necrosis and increased rates of complete tumor response.

To date there are no randomized controlled trials comparing TACE to b-TACE in terms of oncological response; moreover, some retrospective studies reported conflicting results. [5] Ogawa et a l[6] and Irie et al. [7] showed a better tumor response for b-TACE performed with Lipiodol in comparison to Lipiodol TACE performed with a standard catheter. Maruyama et al. [8], on the other hand, failed to demonstrate a difference in tumor control between the two techniques.

The literature regarding the use of this balloon micro-catheter in combination with Drug Eluting microsphere (DEM-TACE) is scarce. To our knowledge, only two studies reported the use of DEM-TACE with a balloon micro-catheter [9, 10] with an objective response of 90% and 100%, respectively. There is currently no evidence on which patients should be offered b-TACE, particularly when the procedure is performed with DEM. This is of particular relevance because the patients included in the BCLC B stage may have a broad spectrum of disease presentations, which may result in lower effectiveness of catheter-based treatments; for example, large tumors (> 50 mm), multiple tumors (> 3) and elevated baseline α-fetoprotein level are all associated with failure to achieve a complete response [11–13]. This is extremely important to understand, since HCC patients with initial complete response after TACE have the longest overall survival, in comparison to other mRECIST response categories [11].

The purpose of our work was to retrospectively analyse in a case–control, retrospective, single center study the results obtained in two groups of HCC patients who underwent catheter based treatment with drug eluted microsphere with a standard micro-catheter and with the use of a balloon micro-catheter (DEM-TACE versus b-TACE).

The primary outcome was to compare results in patients treated with b-TACE and DEM-TACE, in terms of oncological response, and time to recurrence (TTR) after complete response. The secondary outcome was to compare differences in terms of safety profile between the two techniques including post-procedural changes of liver function tests, post-embolic syndrome (PES) and incidence of adverse events.

## Methods

This study was approved by the ethical review board of our Institution. Informed consent for the procedure and for anonymized publication of non-sensitive data was obtained from all individual patients.

This is a case–control, retrospective, single center study.The data of 159 consecutive patients with 248 LI-RADS-5 HCC tumors managed in our tertiary center for liver cancer treatment between January 2015 and March-2019 were reviewed. All TACE indications were discussed at the multidisciplinary tumor board comprising a transplant surgeon, an interventional radiologist, body radiologist and a hepatologist, according to the Quality Improvement Guidelines for Hepatic Transarterial Chemoembolization of the CIRSE [12].

Inclusion criteria were: Child–Pugh score up to B8, Barcelona Clinic Liver Cancer (BCLC) stage up to B,not eligible for curative treatments (surgical resection or percutaneous ablative treatments). Patients presenting with Child–Pugh > B8, BCLC stage C, portal vein thrombosis (defined as the complete or partial obstruction of blood flow in the portal vein, due to the presence of a chronic, acute or neoplastic thrombus), extrahepatic secondary lesions, and high-flow arterioportal or arteriovenous shunts, previous systemic treatments, platelet count < 50,000, and bilirubin level > 3 mg/dL, were not considered suitable for the procedure.

Ten patients who underwent TACE with degradable starch microsphere were excluded. The final study population included 149 patients with 226 HCC. Twenty-two patients (35 HCC tumors; median of 1.6 tumor/patient) were treated with b-TACE (DEM TACE with balloon occlusion) while 127 patients with 191 HCC tumors (median of 1.5 tumors per patient) received standard catheter DEM-TACE without balloon occlusion. Patients’ demographic and clinical characteristics are reported in Table 1.

**Table 1:** Demographic characteristics

	DEM-TACE	B-TACE	*p*
Patient number; nodule number	N:127; N:191	N: 22; N: 35	
Age, year (mean value ± SD)	68.6 ± 10.9	65.9 ± 13.8	0.28
Sex (M/F)	112/15	19/3	0.8
Child pugh N (%)			0.9
A5	55 (43.3%)	11 (50.0%)	
A6	24 (18.9%)	4 (18.2%)	
B7	38 (29.9%)	5 (22.7%)	
B8	10 (7.9%)	2 (9.1%)	
BCLC N (%)			
A	84 (66.1%)	10 (45.5%)	0.06
B	43 (33.9%)	12 (54.5%)	
Etiology: N (%)			0.78
HCV	66 (52%)	9 (41.0%)	
HBV	22 (17.3%)	4 (18.2%)	
Alcohol related cirrhosis	17 (13.4%)	5 (22.7%)	
Cryptogenetic cirrhosis	14 (11%)	3 (13.6%)	
NASH	8 (6.3%)	1 (4.5%)	
MELD: (mean value ± SD)	9.9 ± 2.0	10.0 ± 2.3	0.82
MELDNa: (mean value ± SD)	10.8 ± 2.6	10.8 ± 2.6	0.91
AFP serum level μg/L (median IC95%)	27.5 (1.1–3971)	6.4 (0.7–2599.0)	0.65
Indications for TACE			0.7
Downstaging	20 (15.7%)	3 (13.6%)	
Bridging	50 (39.4%)	7 (31.8%)	
Palliative	57 (44.9%)	12 (54.6%)	

DEM-TACE drug eluting embolics trans-arterial chemoembolization; B-TACE balloon occluded trans-arterial chemoembolization; SD standard deviation; M Male; F Female; BCLC Barcelona clinic liver cancer; HCV Hepatitis C virus; HBV Hepatitis B virus; NASH Non-alcoholic steatohepatitis; MELD Model for End-Stage Liver Disease; AFP α- fetoprotein

All DEM TACE procedures from January 2015 to April 2018 were performed without the use of a balloon micro-catheter for temporary arterial occlusion. The balloon micro-catheter was available at our Institution from April 2018. Considering that there are no recommendations or guidelines for using a balloon micro-catheter for temporary arterial occlusion during DEM-TACE, the decision to use it was left to the Interventional Radiologist preference at the time of the procedure. The embolization protocol at our institution (see following paragraph) was standardized since January 2015.

### DEM-TACE and B-TACE technique

All procedures were performed via femoral access by two experienced Interventional Radiologist (experience > 10 years). After positioning a 4F angiographic catheter in the common/proper hepatic artery, a detailed tumor’s feeder map was performed by digitally subtraction angiography and dual-phase cone beam CT.

After careful identification of the tumor feeders, super-selective catheterization was performed with a 2.7 F micro-catheter (Progreat; Terumo Europe NV, Leuven, Belgium) for DEB-TACE and with a 2.8 F balloon micro-catheter (Occlusafe, Terumo Europe NV, Leuven, Belgium) for B-TACE [10].

The embolization protocol used, for both B-TACE and DEM-TACE, was highly standardized since January-2015. The protocol consisted, as previously reported[14], in a sequential embolization, starting with 100 ± 25 μm PEG microspheres, immediately followed by a second embolization with 200 ± 50 μm, PEG microspheres when needed.

The technical embolization endpoint differs in the two procedures: for DEB-TACE was flow stasis considered as stasis for 10 heartbeats. If stasis was achieved with the injection of 100 μm ± 25 particles, the adjunctive injection of 200 μm ± 50 microspheres was not performed. For b-TACE, the endpoint was different, due to the presence of the inflated balloon micro-catheter that impaired the assessment of flow stasis. Therefore, for this procedure, we used a composite endpoint: upstream reflux of microspheres despite balloon inflation, visualization of vascular anastomosis that could determine potential non-target embolization and manual perception of resistance to the injection of the microspheres [10].

### Follow-up imaging

Imaging follow-up was performed using either contrast enhanced multi-detector computed tomography (MDCT) or contrast enhanced magnetic resonance imaging (CE-MRI) with the use of hepatobiliary contrast agents, according to our institutional protocol (follow-up at 1 month, 3 months and after that every 3–6 months). The response was evaluated according to mRECIST criteria by a radiologists with > 20 years’ experience in CT/MR body imaging as follow: Complete Response (CR) was considered as disappearance of any intra-tumoral arterial enhancement in all target lesions; Partial Response (PR) as a decrease > 30% in the sum of diameters of viable target lesions (taking as reference the baseline sum of the diameters of target lesions); Stable disease (SD) as any cases that do not qualify for either PR or progressive disease (PD), and PD as an increase of at least 20% in the sum of the diameters of viable target lesions (taking as reference the smallest sum of the diameters of viable target lesions recorded since treatment started). Objective response is defined as CR + PR rate; disease control (DC) is defined as CR + PR + SD rate [15, 16].

### Study outcomes and potential confounders

The primary outcome was to compare the oncological results according to mRECIST criteria for patients treated with b-TACE vs DEB-TACE, in terms of oncological response and TTR after complete response. The TTR was calculated at the 1-year follow-up check-point.

Hepatic function of the patients and radiological tumors’ characteristics were potential confounders. Therefore, differences in hepatic function (summarized in Table 2) and radiological tumors’ characteristics (summarized in Table 3) between the two cohorts were considered as co-variants in the statistical analysis only if statistically different; in particular tumor size, which is considered the most important predictive factor for TACE outcome [12].

**Table 2:** Comparison of laboratory values

Laboratory analysis	DEM-TACE				B-TACE				
	Pre	Post	Fold	*p*	Pre	Post	fold	*p*	p^∞^
AST (IU/L) (median CI 95%)	36.0 (30.5 – 43.1)	52.0 (40.5- 65.2)	1.2 (1.1–1.3)	0.0009	38.0 (24.0–50-0)	62.5 (42.4–129.5)	1.2 (0.9–2-1)	0.001	0.84
ALT (IU/L) (median CI 95%)	31.0 (23.5–39-5)	41.5 (30.0–53-1)	1.1 (1.0–1.2)	0.01	24.0 (18.8–45-1)	57–0 (33.0–76-6)	1.3 (0.6–1-1)	0.001	0.72
ALP (IU/L) (median CI 95%)	112.0 (98.9–118-4)	106.5 (99.7–116-2)	0.9 (0.9–1.0)	0.18	120 (86.3–149.9)	109.0 (91.1–138.7)	0.9 (0.0–1.1)	0.10	0.55
γ-GT (IU/L) (median CI 95%)	76.5 (58.9–96.7)	69.0 (54.3–99.0)	0.9 (0.9–1.0)	0.08	65.5 (30.6–134-0)	63.0 (55.0–118.9)	0.9 (0.7–1.1)	0.85	0.72
Bilirubin total (mg/dL) (median CI 95%)	1.0 (1.0–1.1)	1.2 (0.9–1.4)	1.1 (1.0–1.3)	0.1	1.0 (0.8–1.3)	1.5 (0.8–1.9)	1.2 (0.7–1.3)	0.001	0.75
Bilirubin direct (mg/dL) (median CI 95%)	0.5 (0.4–0.5)	0.5 (0.5–0.7)	1.2 (1.0–1.4)	0.1	0.4 (0.3–0.6)	0.7 (0.4–0.9)	1.2 (0.8–1.4)	0.0005	0.74
Albumin(g/L) (median CI 95%)	38.0 (35.0–40.5)	38.0 (34.4)	0.9 (0.0–0.9)	0.29	38.0 (26.3–41.0)	36.0 (28.5–41.7)	0.8 (0.0–1.0)	0.41	0.54
Platelet (× 10^3/μL) (median CI 95%)	93.0 (72.1–113-0)	90.0 (70.0–107.7)	1.0 (1.0–1.1)	0.52	75.0 (46.6–103.2)	65.0 (42.0–99.2)	1.0 (0.9–1.1)	0.60	1.00
Neutrophil (× 10^9/L) (median CI 95%)	1.6 (1.2–2-1)	3.3 (2.6–3.6)	1.7 (1.4–2.2)	< 0.0001	2.0 (1.7–2.5)	3.6 (2.5–5-7)	2.2 (1.1–3.4)	0.009	0.62
INR (median CI 95%)	1.3 (1.2–1.4)	1.2 (1.2–1.4)	1.0 (1.0–1.0)	0.14	1.2 (1.1–1-3)	1.2 (1.2–1-3)	1.0 (0.8–1.0)	0.80	0.27

^**∞**^Comparison between increasing fold; CI: confidence of interval AST: Aspartate Transaminase; ALT: Alanine Transaminase; ALP: Alkaline phosphatase; GGT: gamma-glutamyl transferase; INR: International Normalized Ratio; PLT: Platelets

**Table 3:** Nodules’ characteristics

Nodule characteristics	DEM-TACE	B-TACE	*p*
Dimension maximum diameter. mm. (median CI 95%)	19.0 (17.0–20.0)	27 (21.6–32.4)	< 0.0001
Mean difference	8.0 mm [CI95% 4.0–12.0])		
Range maximum diameter (min–max)	5.0–89.0	8.0–120.0	
Capsulated (number %)	126/191 (66%)	20/35 (57%)	0.32
Adipose degeneration (number %)	4/191 (2.1%)	0/35 (0%)	0.39
Vascular infiltration (number %)	6/191 (3.1%)	0/35 (0%)	0.29
Blurred margin (number %)	57/191 (29.8%)	11/35 (31.4%)	0.85

DEM-TACE: drug eluting embolics trans-arterial chemoembolization; B-TACE: balloon trans-arterial chemoembolization; CI: confidence of interval

The secondary outcome was to compare differences in terms of safety profile between the two techniques including modifications of post-procedural liver function test, occurrence of post-embolic syndrome (PES) and adverse event. PES was defined as fever and/or nausea and/or pain presenting up to 48 h after the procedures[10]. Adverse events (AEs) were evaluated according to the Common Terminology Criteria for Adverse Events (CTCAEv5) [17].

### Statistical analysis

The Kolmogorov–Smirnov Z test was used to assess normality distribution for all variables tested. Continuous normal variables were expressed as mean ± standard deviation. Continuous non-normal variables were expressed as median and confidential interval (CI) 95%. Oncological response was compared using chi-square test at three time points (1 months, 3–6 months, and 9–12 months) on nodule-based analysis (Bonferroni’s correction for post-hoc analysis). For matching pre and post laboratory analysis, the Student T test and the Wilcoxon rank-sum test were used as appropriate according to distribution. A logistic regression was performed for analyzing the impact of hepatic status (MELDNa), gender, age, biochemical tumor spread (AFP), radiological tumor impact (DM max) and presence of micro-balloon catheter on objective response a 9–12 months. For comparing laboratory analysis (in fold modification) and oncological response, between DEM-TACE and B-TACE, Student T test and a Mann–Whitney test were used as appropriate. Chi-square test was used for likening adverse events between the two groups. The PFS was evaluated with Kaplan–Meier curve and Cox’s regression using as tumor dimensions as covariate. Statistical analysis was performed, and the graph was plotted using MedCalc 18.2.1 (MedCalc Software bvba, Ostend, Belgium). P values < 0.05 were considered statistically significant, and all P values were calculated using a two-tailed significance level.

## Results

The study cohort was composed of 149 patients with 226 HCC tumors (B-TACE vs DEM-TACE, 22 vs 127 patients, 35 vs 191 HCC tumors, respectively).

The only statistical difference variable between b-TACE and DEM-TACE cohorts was the median maximum diameter of HCC tumors treated in the B-TACE group compared to DEM-TACE arm (27.0 mm [CI 95% 21.6–32.4] vs 19.0 mm [CI 95% 17.0–20.0]; p < 0.0001; median difference: 8.0 mm [CI95% 4.0–12.0]). All the other tumor and clinical characteristics where similar in both groups (see Tables 1 and 3 for details).

### Oncological results

**Fig. 1:**
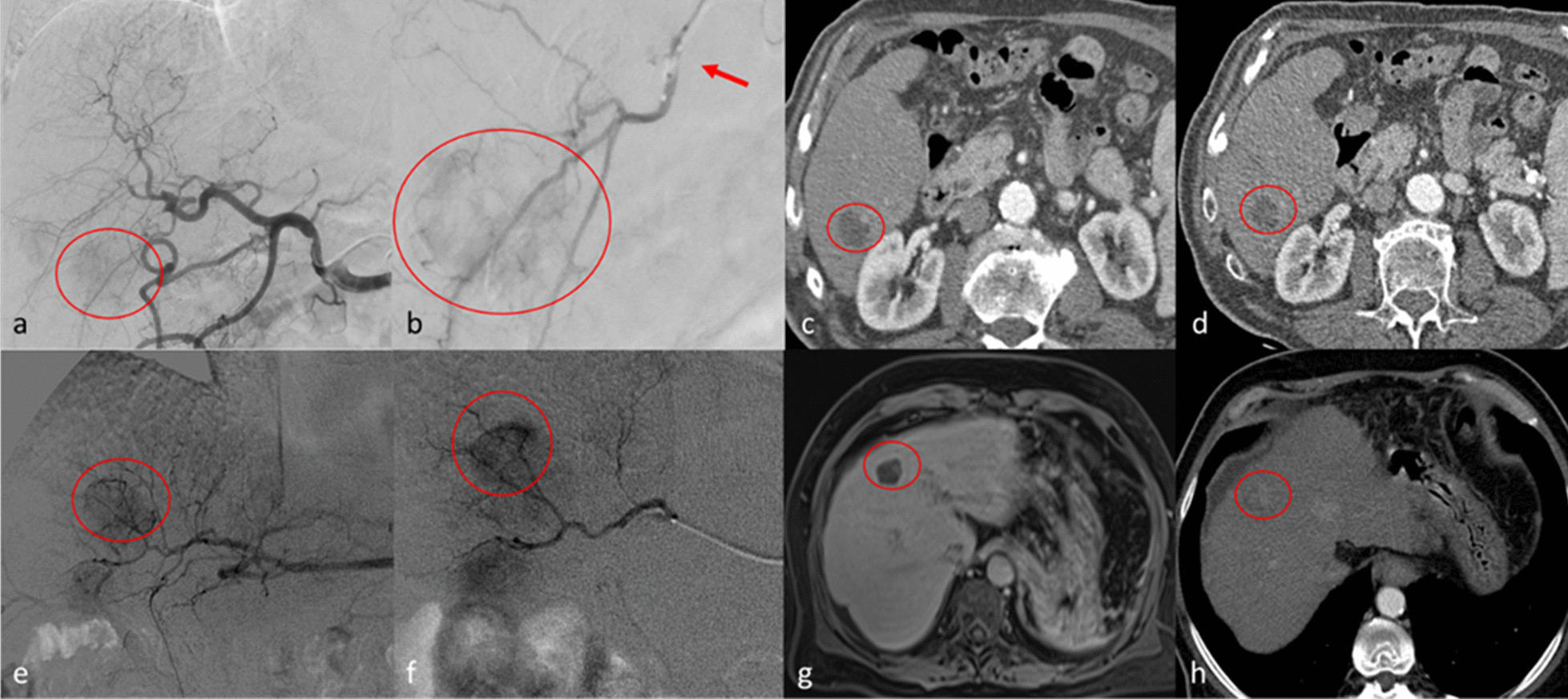
Top row. Clinical case of a 54 years old male with hepatocellular carcinoma [HCC] (diameters: 25 × 23 mm) in segment 6. **a** digital subtraction angiography (DSA) obtained with injection from common hepatic artery demonstrates a hyper vascular tumor (circle); **b** selective DSA with inflated balloon micro-catheter (arrow) confirms the HCC (circle). **c**, **d** show the arterial phase of contrast enhanced computed tomography which demonstrate complete response at 1 month (**c**) and persisting complete response at 6 months (**d**). Bottom row. Clinical case of a 61 years old female with HCC (diameters: 22 × 21 mm) in the segment 4. **e**, **f** DSA from common hepatic artery and super-selective DSA with micro-catheter, respectively, demonstrate the HCC (circle). **g** MR imaging follow-up in hepatobiliary phase shows complete response at 1 month (circle); **h** at 6 months follow-up, computed tomography in arterial phase shows only a partial response

Per-nodule analysis demonstrated no significant differences in the oncological response at all time points (1, 3–6 and 9–12 months) for both treatments, with the exception of Objective response rate at 9-12 months. In particular: Complete response was [b-TACE vs DEM-TACE] 14/35 (40.0%) vs 81/191 (42.4%) at 1 month, 13/29 (44.8%) vs 62/136 (45.6%) at 3–6 months and 13/19 (68.4%) vs 45/89 (50.6%) at 9–12 months (*p* > 0.05). Objective response was similar at 1 and 3–6 months between b-TACE vs DEM-TACE [23/35 (65.7%) vs 119/191 (62.3%), 21/29 (72.4%) vs 78/136 (57.4%) (*p* > 0.05), respectively]. On the contrary, at 9–12 months, it was significantly higher in b-TACE subgroup than DEM-TACE (15/19 [78.9%] vs 48/89 [53.9%], *p* = 0.05) (see Table 4 for detailed data) (Fig. 1.). Stable disease was significantly higher for DEM-TACE vs b-TACE at 9–12 months (30.3% vs 0%, *p* = 0.0006), however disease control remained not statistically significant due to compensation of Complete and Objective Response in b-TACE group. No statistical significancy were found regarding the presence of the balloon micro-catheter in the logistic regression for objective response at 9–12 months (OR 1.70 [CI95% 0.32–8.96], *p* = 0.53) and for the remaining parameters (MELDNa, gender, AFP, age and max diameter; OR 0.82 [CI95% 0.66–1.03]; 4.27 [CI95% 0.78–23.4]; 1.00 [CI95% 0.99–1.00]; 1.01 [CI95% 0.95–1.06]; 1.01 [CI95% 0.97–1.06]).

**Table 4:** Oncological results

	Per-nodule		
1 months			0.33
Complete response	42.4% (81/191)	40.0% (14/35)	0.79
Partial response	19.9% (38/191)	25.7% (9/35)	0.44
Stable disease	34.6% (66/191)	25,7% (9/35)	0.31
Progressive disease	3.1% (6/191)	8.6% (3/35)	0.13
Objective response	62.3% (119/191)	65.7% (23/35)	0.70
Disease control	96.8% (185/191)	91.4% (32/35)	0.13
3–6 months			0.14
Complete response	45.6% (62/136)	44.8% (13/29)	0.94
Partial response	11.8% (16/136)	27.6% (8/29)	0.03°
Stable disease	30.1% (41/136)	20.7% (6/29)	0.31
Progressive disease	12.5% (17/136)	6.9% (2/29)	0.39
Objective response	57.4% (78/136)	72.4% (21/29)	0.13
Disease control	87.5% (119/136)	93.1% (27/29)	0.39
9–12 months			0.03
Complete response	50.6% (45/89)	68.4% (13/19)	0.24
Partial response	3.4% (3/89)	10.5% (2/19)	0.18
Stable disease	30.3% (27/89)	0% (0/19)	0.006
Progressive disease	15.7% (14/89)	21.1% (4/19)	0.57
Objective response	53.9% (48/89)	78.9% (15/19)	0.05
Disease control	84.3% (75/89)	78.9% (15/19)	0.50

CI: confidence interval; DEM-TAC: drug eluting embolics trans-arterial chemoembolization; B-TACE: balloon trans-arterial chemoembolization; LAF: last available follow-up. ° The Bonferroni’s correction in this case significantly addressed p at 0.0125 (0.05/4)

**Fig. 2:**
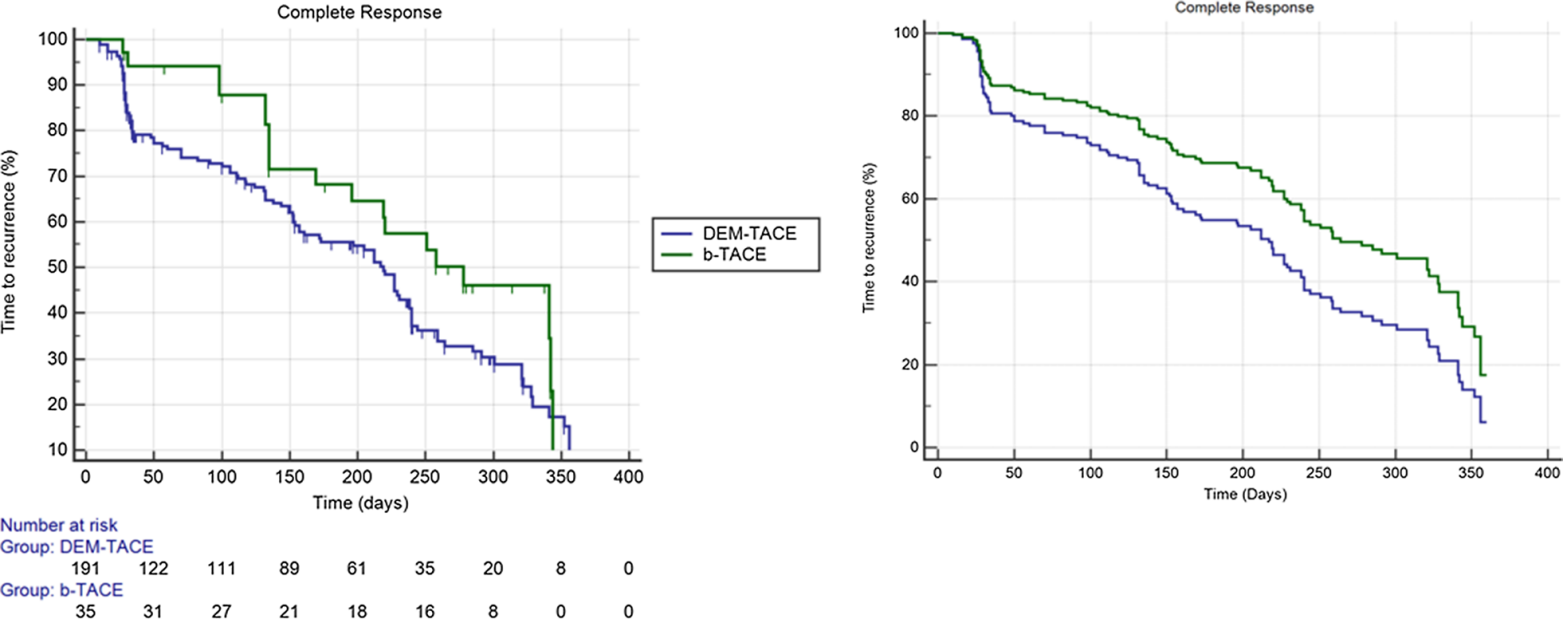
Kaplan Meier analysis of time to recurrence for complete response at 9–12 months follow-up (**a**). **b** Showed the Cox-regression analysis weighted for the presence/absence of micro-balloon catheter and tumor dimension confirming the better trend of time to recurrence for b-TACE comparing to DEM-TACE

The median follow-up time was 143 days (CI95% 132.0–154.0), which is higher in b-TACE comparing with DEM-TACE (162.5 days [CI95% 134.2–227.9] vs 132.0 days [98.6–154.0], *p* = 0.03). Only 108/226 (47.8%) reached the timeframe of 9–12 months follow-up. There was a trend for better median TTR for b-TACE vs DEM-TACE for complete response at 9–12 months (278.0 days [196.0–342.0] vs 219.0 days [161.0–238.0], odd ratio [OR] 0.68 [0.4–1.0], *p* = 0.10). (Fig. 2.). This higher trend of TTR was confirmed by Cox-regression with a relative risk of event of 0.63 (CI95% 0.38–1.04, *p* = 0.07) for the presence of the micro-balloon catheter and of 1.0 (CI95% 0.99–1.02, *p* = 0.46) for tumors’ dimension (Fig. 2.).

### Safety profile

Adverse events were observed without significant difference between B-TACE and DEM-TACE (grade 3: 1/22 [4.5%] vs 3/127 [2.4%] and grade 2: 4/22 [18.1%] vs 20/127 [15.7%], *p* > 0.05, respectively). In particular, a pseudo-aneurysm was recorded for a B-TACE procedure, and an intra-hepatic artery dissection and acute renal insufficiency requiring dialysis were observed in two DEM-TACE procedures. PES was experienced by 8/22 (36.4%) of B-TACE patients and 32/127 (25.2%) of DEM-TACE (*p* = 0.28).

Regarding laboratory values, no statistically significant differences were found between the two interventions for all parameters considered at post-procedural evaluation (see Table 2. For details).

## Discussion

The analysis of our data shows that b-TACE has a trend of better oncological response over DEM-TACE. This was supported by an improvement in long term oncological response (Objective response 78,9% [b-TACE] vs 58,9% [DEM-TACE] at 9–12 months, *p* = 0.05) and a longer Time to Progression (TTR) after Complete Response over standard non-occluded DEM-TACE. This is of particular relevance, considering that b-TACE cohort included larger size tumors (mean diameter: 27 mm [b-TACE] vs 19 mm [DEM-TACE]), and that the adverse events rate were comparable between two techniques.

Transarterial chemoembolization (TACE) represent the standard of care for intermediate HCC. Its aim is to locally deliver to the target lesion the maximum amount of drugs and non-re-absorbable microspheres, thus permitting local tumor control. Recently b-TACE, thanks to its ability to redistribute flow towards lower resistance vascular territories and allowing a pressure-gradient driven embolization, has demonstrated to be capable to improve drug delivery to target lesion[4]. This technical benefit should theoretically enhance the ability to locally control tumor growth. Despite this, literature evidence on oncological response of b-TACE over standard non occluded TACE is controversial[5, 7, 8, 18]

In order to evaluate the adjunctive value of b-TACE we retrospectively evaluated the results of patients treated in our institution with b-TACE and compared them with an historical cohort treated with non-occluded DEM-TACE. b-TACE and DEM-TACE were performed by the same team under dual phase CBCT guidance i.e..: better tumor/feeders visualization) [19], with rigorous standardization of the embolization procedure (sequential embolization with 100 and 200 microns particles[14]), being the only technical variable the balloon micro-catheter employment. Moreover by comparing our study to the one reported by Irie et al.[7], the only that compared superselective b-TACE to superselective TACE (both performed with Lipiodol emulsion), emerges several differences. First, the embolic agent is different; second we enrolled a larger control population; third, mean diameter of the treated nodule are different, in particular: in our study treated nodule are smaller in both group (b-TACE 27 mm; TACE 19 mm) if compared to the Irie’s one (b-TACE 39 mm; TACE 40 mm); finally nodule treated with TACE in the Irie’s series were not naïve. All these variables render direct comparison of the study results limited.

With regards to the clinical response, b-TACE demonstrated an improvement in oncological response at 9–12 months (Objective response 78.9% [b-TACE] vs 58,9% [DEM-TACE], *p* = 0.05), whereas at other time points (1, 3–6 months) we didn’t observed statistically different response rates. Moreover, B-TACE cohort’s tumor had a larger median diameter compared to DEM-TACE (8.0 mm [CI95% 4.0–12.0]). This is particularly relevant considering that tumors’ size is one the major factors influencing oncological response after TACE (odds ratio per centimeters [OR] 2.85, *p* = 0.002) [20] and overall response (OR) is strongly correlated with positive clinical outcomes (recurrence rate: 35.8% [non-responder and tumors > 3 cm] vs 11.9% [responder and tumors > 3 cm]) [21]. Although, the logistic regression using objective response at 9–12 months as outcome showed no significancy for the presence of the balloon micro-catheter.

B-TACE had a trend for higher TTR after an initial complete response vs DEM-TACE at 1-year, confirmed also by the Cox-regression analyses weighted for the presence of micro-balloon catheter and tumors’ diameter. This should be explained by several reasons: i) B-TACE procedures were performed by positioning the device proximal to all tumor’s feeders, thus less selective than DEM-TACE procedures, therefore allowing for better pharmacological coverage of the area immediately surrounding the HCC tumors; ii) complete response tumors received a more targeted dose of drug and particles due to pressure gradient driven embolization that improves distribution to the tumoral vasculature [5]. This result is of particular importance considering that a complete response after first chemoembolization is still the most robust predictor for long-term favorable outcome (Overall Survival) in hepatocellular carcinoma according to Kim et al.[11]. In addition, it could play a role in maintaining patients in active transplantation list for longer time.

No differences were observed between B-TACE and DEM-TACE in terms of AEs. Of note, the grade 3 AEs (pseudo-aneurysm) observed in the B-TACE subgroup occurred during the learning curve of balloon micro-catheter usage (within the first five cases) [10, 22]. It is to be noted that also during DEM-TACE procedures grade 3 hepatic artery injury (defined as occlusions) occurred [8/205 (3.9%) after 2 sessions of TACE] as reported by Suh et al.[23]. Regarding PES, both groups had a similar percentage of incidence (36.4% and 25.2%), and this is in accordance with the existing literature regarding DEM-TACE (range 24.7%-75%)[24]. Both sub-groups of this study experienced a transient rise of AST, ALT, and neutrophils, and no single parameter increased more than 1.5 fold (CTCAEv5 grade 1). This findings are comparable with published literature[24]. Moreover B-TACE patients experienced a slight increase of bilirubin and direct bilirubin (fold: 1.2 (0.7–1.3) and 1.2 (0.8–1.4), respectively), reflecting a possible major impact of the embolization performed with the micro-balloon on the biliary tree. In fact, the peri-biliary plexus is one of the intrahepatic collateral pathways that open after balloon inflation[5]. For this reasons several authors [8] [25], advised extra caution when using a balloon micro-catheter to perform-TACE in patients with bile duct dilatation[5].

This study presents some limitations. First, the nature of the study is retrospective and observational without randomization. Second, groups were not homogenous, though this limitation was overcome by weighting differences as co-variate in statistical analysis.

## Conclusions

B-TACE had a better objective response at 9–12 months and higher TTR after CR at 1-year in comparison to DEM-TACE, with a similar AEs rate, in patients presenting with larger tumors. These findings suggest a potential advantage of B-TACE for patients with larger tumors. If these results will be confirmed in on-going large-scale studies, B-TACE may be offered as a safe and effective alternative to current standard catheter TACE in selected patients.

## Data Availability

The datasets used and/or analysed during the current study are available from the corresponding author on reasonable request.

## Abbreviations

TACE: trans-catheter-arterial chemoembolization
b-TACE: balloon-occluded trans-catheter arterial chemoembolization
DEM-TACE: drug eluting microsphere trans arterial chemoembolization
HCC: hepatocellular-carcinoma
mRECIST: modified-response-evaluation-criteria-in-solid-tumor
TTR: time to recurrence
BCLC: barcelona clinic liver cancer
PES: post-embolic syndrome
MDCT: multi detector computed tomography
CE-MRI: contrast enhanced magnetic resonance imaging
CR: complete response
PR: partial response
SD: stable disease
PD: progressive disease
AEs: adverse events
CTCAEv5: common terminology criteria for adverse events
