# Correction to: Balloon occluded TACE (B-TACE) vs DEM-TACE for HCC: a single center retrospective case control study

**DOI:** 10.1186/s12876-021-01861-y

**Published:** 2021-07-09

**Authors:** Pierleone Lucatelli, Gianluca De Rubeis, Bianca Rocco, Fabrizio Basilico, Alessandro Cannavale, Aurelio Abbatecola, Pier Giorgio Nardis, Mario Corona, Stefania Brozzetti, Carlo Catalano, Mario Bezzi

**Affiliations:** 1 Vascular and Interventional Radiology Unit, Department of Diagnostic Service, Sapienza University of Rome, Viale Regina Elena 324, 00161 Rome, Italy; 2 Gastroenterology Division, Department of Clinical Medicine, Sapienza University of Rome, Rome, Italy; 3 Pietro Valdoni Surgery Department, Sapienza” University of Rome, Rome, Italy

## Correction to: BMC Gastroenterol (2021) 21:51 https://doi.org/10.1186/s12876-021-01631-w

Following publication of the original article [1], the authors identified an error in the author name of Stefania Brozzetti.

- The incorrect author name is: Stefania Brozetti.
- The correct author name is: Stefania Brozzetti.

The author group has been updated above and the original article [1] has been corrected.
